# Metagenomic Applications to Herbivore Gut Microbiomes: A Comprehensive Review of Microbial Diversity and Host Interactions

**DOI:** 10.3390/ani15202938

**Published:** 2025-10-10

**Authors:** Jinjin Wei, Lin Wei, Abd Ullah, Mingyang Geng, Xuemin Zhang, Changfa Wang, Muhammad Zahoor Khan, Chunming Wang, Zhenwei Zhang

**Affiliations:** 1 College of Agriculture and Biology, Liaocheng University, Liaocheng 252000, Chinazahoorkhan@lcu.edu.cn (M.Z.K.); 2 Yili Kazak Autonomous Prefecture Livestock General Station, Yili 835000, China

**Keywords:** herbivores, gut microbiota, metagenomics, ruminants, non-ruminants, microbial diversity, short-chain fatty acids, production performance, probiotics, nutrient metabolism

## Abstract

**Simple Summary:**

This comprehensive review demonstrates how metagenomic technologies have revolutionized understanding of herbivore digestive systems through culture-independent analysis of gut microbial communities. The research reveals fundamental differences between ruminant foregut fermentation strategies and non-ruminant hindgut approaches, with distinct microbial compositions dominated by *Firmicutes* and *Bacteroidetes*. Key findings show that specific microbial taxa directly correlate with feed efficiency, growth performance, and animal health outcomes. The authors identify critical research gaps in sampling methodologies and functional validation while highlighting promising applications of probiotics, prebiotics, and targeted interventions for optimizing livestock production and reducing environmental impacts through improved microbial management strategies.

**Abstract:**

Herbivorous animals rely on complex gastrointestinal systems and microbial communities to efficiently digest plant-based diets, extract nutrients, and maintain health. Recent advances in metagenomic technologies have enabled high-resolution, culture-independent analysis of gut microbiota composition, functional potential, and host–microbe interactions, providing insights into microbial diversity across the herbivore digestive tract. This review summarizes key findings on the gastrointestinal microbiota of herbivores, focusing on ruminant foregut and non-ruminant hindgut fermentation. Ruminants like cattle, sheep, and goats host microbiota enriched with fibrolytic and methanogenic microbes that facilitate fiber degradation and volatile fatty acid production, contributing significantly to energy balance. In contrast, non-ruminants such as horses and rabbits rely on hindgut fermentation, with distinct microbial taxa contributing to carbohydrate and protein breakdown. The review further explores how specific microbial taxa, including *Prevotella*, *Fibrobacter*, and *Ruminococcus*, correlate with improved feed efficiency and growth performance, particularly in ruminants. Additionally, the roles of probiotics, prebiotics, and symbiotics in modulating gut microbial composition and enhancing productivity are discussed. Despite significant advances, challenges remain in microbial sampling, functional annotation, and understanding the integration of microbiota with host physiology. The review emphasizes the potential of metagenomic insights in optimizing herbivore gut microbiota to improve feed efficiency, health, and sustainable livestock production.

## 1. Introduction

Herbivores are animals that mainly rely on plants as their food source to obtain energy and nutrients necessary for their life activities. Herbivores are an important component of the energy and material circulation system [1]. Herbivores can be classified into ruminants, such as cattle, sheep, and goats, which rely on foregut fermentation, and non-ruminants, such as horses and donkeys, which primarily rely on hindgut fermentation [2]. The main difference between herbivores and non-herbivores lies in the structure of the digestive tract system and its interaction with the microbial flora [3]. The digestive systems of herbivores have evolved to accommodate the breakdown of complex plant materials like cellulose, which they cannot digest on their own [4]. Their gut microbiota, consisting of a diverse community of microorganisms, play a pivotal role in fermenting these materials and producing energy sources such as short-chain fatty acids [5]. However, they have evolved specialized digestive systems, like the ruminant rumen and the equine cecum, to ferment complex plant carbohydrates into volatile fatty acids that support growth and energy balance [6,7]. Unlike ruminants with their complex multi-chambered stomachs, donkeys and horses rely on hindgut fermentation, making their cecal and colonic microbiota critical for nutrient digestion and overall health. Metagenomic technology has revolutionized our understanding of microbial communities in non-ruminant herbivores, particularly in equines, which serve as important working animals and alternative livestock species since their domestication. Recent advances in metagenomic sequencing, including high-throughput 16S rRNA gene sequencing and whole-genome shotgun metagenomics, have enabled comprehensive characterization of the donkey microbiome across different anatomical sites and physiological states. Studies employing metagenome-assembled genomes (MAGs) have provided unprecedented insights into the functional capabilities of donkey hindgut microbiota, revealing complex metabolic pathways involved in fiber degradation and volatile fatty acid production [8]. Their gut microbiota is diverse, contributing to nutrient absorption and immune regulation, but its composition is influenced by diet, environment, developmental stage, host health, and genetics [9]. The gastrointestinal microbiota plays a pivotal role in the digestion and metabolism of herbivorous animals [10,11].

Recent advancements in metagenomic sequencing have revolutionized the study of microbial communities, enabling comprehensive analysis of their composition and functional potential [12,13]. Metagenomics overcomes the limitations of traditional culture methods by bypassing the need for isolated cultures, allowing direct analysis of the genetic material present in environmental samples. This approach captures the genetic information from a diverse range of microorganisms, including those that cannot be cultured by conventional techniques [14]. Through modern sequencing technology, genomic information in samples can be obtained rapidly and on a large scale [15]. Metagenomics has expanded microbial research beyond culturable strains, allowing the study of unculturable microorganisms and their interactions with hosts. It enables identification of microbial composition, genetic and functional profiles, evolutionary relationships, and environmental interactions, with advances like binning technology improving genome classification [16,17]. The exploration of gastrointestinal microbiota in herbivores has revealed complex interactions between microbial communities and host physiology [18]. Furthermore, metagenomic approaches have illuminated spatial variations in microbial composition across different intestinal segments, demonstrating distinct community structures between foregut and hindgut regions, as well as between liquid and adherent phases within the caeco-colic ecosystem [19,20]. Beyond the digestive tract, metagenomic surveys have characterized microbial communities in other body sites, including oral, skin, and rectal microbiota, showing dynamic changes during critical life stages such as weaning and gestation [21,22,23]. Microbes such as bacteria, archaea, protozoa, and fungi contribute not only to the breakdown of complex plant polysaccharides but also to the synthesis of essential nutrients, vitamins, and bioactive compounds [24]. Moreover, the microbial ecosystem influences host immune function, intestinal development, and resistance to pathogens, highlighting its integral role in maintaining overall animal health [11].

The development of sequencing technology has significantly enhanced the integrity and accuracy of genome assembly, while the development of bioinformatics tools has improved the ability to annotate microbial functional genes [25]. In recent years, the development of metagenomics has promoted the research on microorganisms at the macro level, providing a new perspective for understanding the role of microorganisms in host nutrition, health and environmental adaptation [13,26]. This review focuses on the application of metagenomics in studying herbivore gastrointestinal microbiomes, using techniques like shotgun sequencing, metatranscriptomics, and metaproteomics to analyze microbial diversity, metabolic pathways, and host–microbe interactions, highlighting their role in regulating microbial efficiency in the host [27,28,29].

Despite extensive microbiome research in individual herbivore species, previous reviews have been neither sufficiently in-depth nor species-specific in their analyses, with non-ruminants—particularly equines—receiving considerably less comprehensive coverage. This gap underscores a critical need for systematic comparative analyses that integrate metagenomic technologies with production outcomes across diverse digestive system architectures. This review addresses several key knowledge gaps that distinguish it from the existing literature. First, while previous reviews have focused primarily on either ruminants or non-ruminants separately, this work provides a systematic comparative framework examining both foregut and hindgut fermentation strategies and their distinct microbial ecosystems. Second, we critically evaluate the methodological challenges in gut microbiota sampling—from invasive rumen cannulation to emerging non-invasive approaches—an aspect often overlooked in existing reviews but essential for standardizing future research protocols. Third, this review uniquely integrates antimicrobial resistance (AMR) patterns within herbivore microbiomes, addressing an emerging concern for both animal health and public safety. Fourth, we synthesize recent advances in multi-omics approaches (metagenomics, metatranscriptomics, metaproteomics, and metabolomics) that enable functional validation beyond taxonomic profiling. Finally, by explicitly linking specific microbial taxa to production performance metrics (feed efficiency, growth rates, and health outcomes), this review provides actionable insights for precision livestock management. These integrated perspectives aim to guide future research toward (1) developing standardized, non-invasive sampling methodologies; (2) establishing functional databases for improved gene annotation; (3) elucidating mechanistic host–microbe interactions under varying environmental and dietary conditions; and (4) implementing microbiota-based interventions for enhanced productivity and sustainability in herbivore agriculture.

## 2. Gastrointestinal Characteristics of Herbivores

During the process of biological development and evolution, due to climate change and the influence of low-availability nutrients in the diet, a large number of herbivores became extinct. However, the interaction between animals and plants enabled some animals to evolve special digestive systems, allowing them to obtain higher nutritional value and provide energy for their bodies, thereby enabling them to survive and develop [30]. Herbivores have evolved well-developed cellulose-breaking functions due to their herbivorous habits, such as ruminants and large single-stomach herbivores. This adaptive feature can help them digest plant-based feeds well and provide energy for their bodies. Herbivores can be classified into ruminants that mainly rely on the rumen for fermentation and non-ruminants that mainly rely on the intestine for fermentation (such as the cecum) based on the site of their decomposition of plant-based feeds. The main difference between ruminants and non-ruminants lies in a series of septal pouch structures in front of their true stomachs [31]. Ruminants have a unique digestive system. Compared with other herbivores, they can make more efficient use of the energy form fibrous plants [32].

According to the anatomical structure of the gastrointestinal tract of ruminants, their digestive system is composed of a multi-compartment stomach structure (rumen, reticulum, omasum, abomasum), small intestine (duodenum, jejunum, ileum), and large intestine (colon, cecum, rectum) [33]. However, not all ruminants have the characteristic four-chambered stomach structure. For instance, camels lack an omasum. The anterior stomach includes the rumen, reticulum, and omasum [34]. It is a special digestive structure that distinguishes ruminants from other animals. It can physically process feed, undergo microbial fermentation, and absorb nutrients, and is a key structure for digesting cellulose. The rumen is the first and most important part of the stomach in ruminants and is rich in bacteria that can digest cellulose, such as *Fibrobacter* [35], *Ruminococcus* [36], *Butyrivibrio* [37], and *Prevotella* [38]. During the digestive process, ruminants, in contrast to non-ruminants, undergo rumination, where they regurgitate and re-chew their feed to break it down into smaller, more digestible particles [39]. This mainly relies on the density sorting mechanism of the anterior stomach [40]; this mechanism can increase the intake of ruminants without affecting digestive efficiency. The abomasum, known as the true stomach, is structurally and functionally similar to other monogastric animals. It can secrete gastric acid and various digestive enzymes to break down proteins and fats, facilitating their absorption and utilization in the intestinal tract.

**Figure 1:**
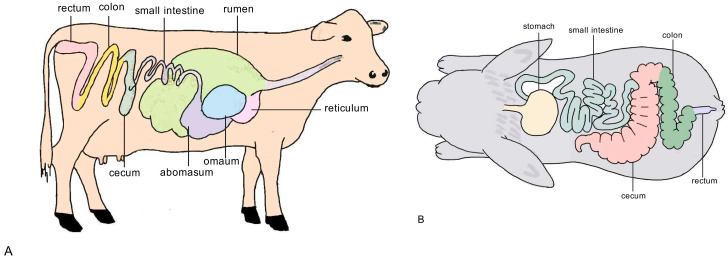
Schematic diagram of the gastrointestinal tracts of representative ruminants and non-ruminants. (**A**) Left side: Ruminant gastrointestinal tract showing the complex, multi-chambered stomach (rumen, reticulum, omasum, and abomasum). (**B**) Right side: Non-ruminant gastrointestinal tract with a simpler stomach structure and reliance on the cecum and large intestine for fermentation.

The digestive tract of non-ruminants is composed of the same parts as that of other animals, including the stomach, duodenum, jejunum, ileum, cecum, colon, and rectum. They do not have a well-developed rumen. The stomach of non-ruminants has similar functions to those of other monogastric animals, but it has a well-developed cecum and colon, which have a fermentation function similar to that of the rumen in ruminants. The bacteria and ciliates inside can break down roughage [32]. Compared to ruminants, non-ruminants have relatively small stomachs that cannot store large quantities of feed. As a result, they require frequent feeding and are adapted to the continuous intake of pasture. The schematic diagram of the gastrointestinal tracts of representative ruminants and non-ruminants is presented in Figure 1.

Ruminants are pre-intestinal fermenters. The rumen can store a large amount of feed and is regarded as a fermentation tank, providing a perfect living environment for the proliferation, development, and metabolic activities of microorganisms. Microorganisms can digest fibrous substances into volatile fatty acids, which are then absorbed by the body, serving as the primary energy source for ruminants. Non-ruminant herbivores rely on hindgut fermentation [41]. With a well-developed cecum, their digestive capacity is limited by the volume of the intestine and the rate at which digesta passes through. The cecum and large intestine act as fermentation chambers, serving as the main sites where microorganisms degrade and ferment cellulose [42]. The volatile fatty acids produced through fermentation are then absorbed and metabolized to provide energy for the host [43]. The gastrointestinal characteristics of ruminants and non-ruminants are summarized in Table 1.

## 3. Research Strategies of Metagenomic Technology in Herbivores

### 3.1. Metagenomic Technologies

The term “metagenome” was first proposed by Handelsman et al. in 1998, referring to the collective genomes of all microorganisms in a specific environment, analyzed as a single genomic unit [71]. Metagenomics technology does not require the microbial isolation and culture by directly extracting total microbial DNA from environmental samples, constructing genomic libraries, and screening for novel functional genes and metabolites. Common approaches include whole-genome shotgun sequencing and amplicon sequencing [72,73]. DNA is fragmented by physical or enzymatic methods, sequenced, assembled, and annotated for species classification and functional potential. Early studies relied on 16S rRNA sequencing to profile microbial diversity, though this method cannot resolve subspecies, strains, or non-bacterial symbionts such as fungi, viruses, and protists [71,74]. High-throughput metagenomic sequencing combined with bioinformatics enables strain-level analysis, functional prediction, host–microbe interaction studies, and exploration of uncultured microbial “dark matter” [75].

Advances in sequencing technologies and bioinformatics have enabled reconstruction of whole microbial genomes from complex communities [76,77]. Short- and long-read sequencing, along with improved algorithms, allows for binning sequences into taxonomic clusters, improving genome completeness [75,78,79]. Long-read sequencing enhances assembly quality by increasing the continuity of metagenomic assembly, which in turn allows for more accurate reconstruction of microbial genomes, making it a crucial tool for analyzing microbial metabolic pathways [80,81,82,83,84]. Integration with multi-omics approaches, including metatranscriptomics and metabolomics, further elucidates the relationship between gene expression and ecological function [85]. Therefore, metagenomics is widely applied in various fields of microbial research, such as agriculture, biology, pollution control, energy, environment and other areas [86]. Advances in sequencing and multi-omics have made metagenomics a powerful tool for reconstructing microbial genomes and understanding their functional roles in complex ecosystems.

Despite these advances, metagenomics faces challenges. Host DNA contamination can compromise microbial resolution, necessitating selective lysis, enzymatic degradation, and enrichment of microbial DNA prior to sequencing [87,88,89,90,91,92,93,94]. Functional annotation is also limited by fragmented assemblies and incomplete reference databases, highlighting the need for improved computational tools and curated genomic resources [95]. The typical metagenomic workflow includes genome enrichment, DNA extraction, library construction, sequencing, gene prediction, and functional expression analysis.

### 3.2. Metagenomic Research Strategies

Metagenomic research strategies are broadly categorized into two principal approaches: (1) sequence-based metagenomics and (2) function-based metagenomics [96,97].

Sequence-based metagenomics employs shotgun sequencing of total environmental DNA to comprehensively characterize the genetic composition and functional potential of microbial communities. This approach involves the random fragmentation of genomic DNA from all microorganisms within a sample, followed by high-throughput sequencing and subsequent bioinformatic analysis to identify taxonomic composition and predict functional gene content.

Function-based metagenomics utilizes activity-driven screening methods to identify specific functional genes or biomolecules of interest. This approach typically involves constructing metagenomic expression libraries in suitable host organisms and screening clones for desired phenotypic traits or enzymatic activities, enabling the direct detection of functional variants independent of sequence homology.

In intestinal microbiota studies utilizing sequence-based approaches, biological samples—including gastric contents, intestinal digesta, or fecal material—are collected for metagenomic DNA extraction. Following DNA fragmentation and sequencing library preparation, raw sequence data undergo quality control procedures to remove low-quality reads and filter host-derived contamination. High-quality reads are then assembled into contiguous sequences and subjected to binning algorithms to reconstruct metagenome-assembled genomes (MAGs). Taxonomic classification of MAGs is performed using reference databases such as the Genome Taxonomy Database (GTDB) and the National Center for Biotechnology Information (NCBI) database, while functional annotation is conducted through mapping against the Kyoto Encyclopedia of Genes and Genomes (KEGG) and Gene Ontology (GO) databases.

However, in addition to KEGG and GO, several important functional annotation databases play crucial roles in gene functional analysis. CAZy and dbCAN are essential for analyzing carbohydrate-active enzymes (CAZymes) [98], while eggNOG and COG are vital for identifying orthologous gene groups, aiding in functional annotation across species [99]. UniRef enhances protein function identification by clustering similar protein sequences [100,101,102]. Tools like eggNOG-mapper v2 assign genes to orthologous groups and integrate annotations from GO, KEGG, and CAZy, facilitating functional inference across diverse organisms [99]. Similarly, dbCAN utilizes the CAZy database for automated CAZyme annotation, highlighting its significance in annotating carbohydrate-active enzymes in microbial genomes [98].

**Figure 2:**
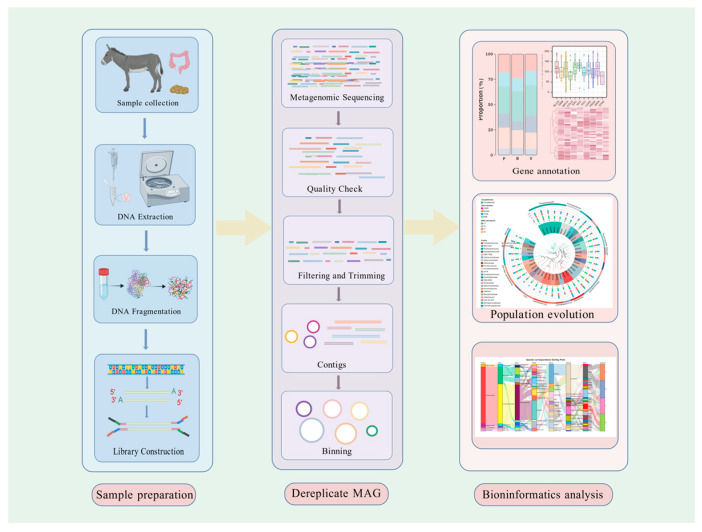
Workflow of metagenomic analysis in herbivores. The diagram outlines the stepwise process starting from sample collection, DNA extraction, and fragmentation, through library construction, sequencing, and quality control, to metagenomic assembly, binning, dereplication, functional and taxonomic annotation, pathway enrichment, and personalized analysis. This workflow illustrates the integration of sequencing technologies and bioinformatics tools in characterizing microbial communities. Created with BioGDP.com [109].

Finally, species richness, gene enrichment, and metabolic pathway analyses are performed to characterize microbial community structure and functional potential [76,103,104,105,106,107,108]. Metagenomic research in herbivores employs a systematic analytical workflow that enables comprehensive characterization of microbial communities through sequential processes spanning DNA extraction, bioinformatics analysis, functional annotation, and customized data interpretation. This integrated approach, as demonstrated in Figure 2, facilitates the elucidation of complex microbiome structures and their ecological roles within herbivorous systems. The implementation of both function-based and sequence-based metagenomic methodologies provides complementary analytical frameworks for the comprehensive assessment of microbial diversity, functional capacity, and metabolic potential within these intricate biological communities.

## 4. Differences Between the Gastrointestinal Microbiota of Ruminants and Non-Ruminants

**Figure 3:**
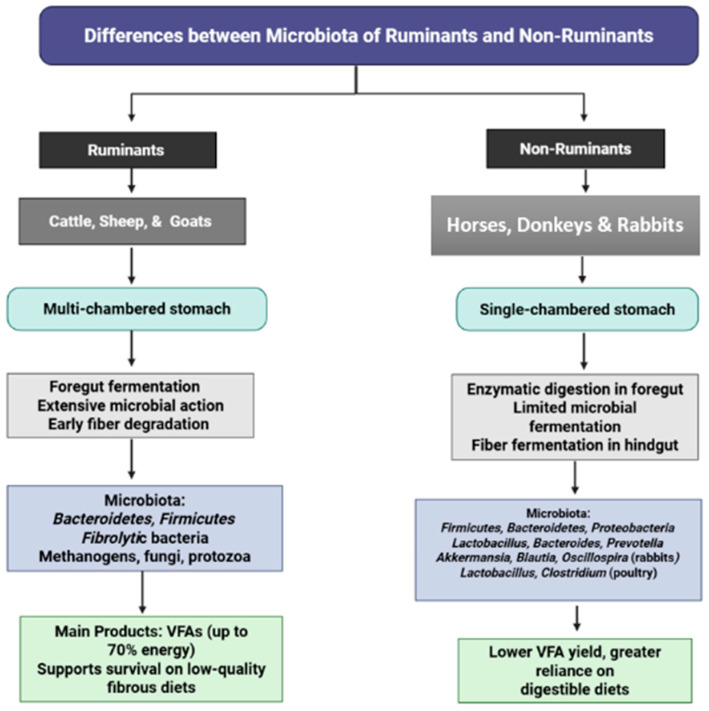
Comparison of gastrointestinal microbiota between ruminants and non-ruminants.

The herbivore digestive system is uniquely adapted to plant-based diets, a characteristic distinctly reflected in the composition and function of its gastrointestinal microbiota. Ruminants, including cattle, sheep, and goats, are foregut fermenters possessing a multi-chambered stomach system (rumen, reticulum, omasum, and abomasum) where microbial communities extensively degrade fibrous plant material before it encounters host digestive enzymes [110,111,112,113,114,115,116]. Beyond bacteria, methanogenic archaea (e.g., *Methanobrevibacter*, *Methanosarcina*), anaerobic fungi, and protozoa contribute synergistically to cellulose and hemicellulose breakdown, producing volatile fatty acids (VFAs) that supply up to 70% of the host’s energy requirements [10,117,118,119]. This specialized microbial ecosystem enables ruminants to efficiently utilize low-quality, high-fiber diets that remain largely indigestible to non-ruminant species. These fundamental compositional differences in microbiota architecture are comprehensively illustrated in Figure 3.

In contrast, non-ruminant herbivores such as horses, donkeys, rabbits, and deer possess single-chambered stomachs where digestion depends primarily on host-derived enzymes, with microbial fermentation occurring predominantly in the hindgut (cecum and colon) [48,120]. Rabbits demonstrate heavy reliance on cecal fermentation and employ cecotrophy to maximize nutrient absorption, exhibiting enrichment of *Akkermansia*, *Blautia*, and *Oscillospira* in soft feces [68,121]. These structural and functional differences necessitate more digestible dietary inputs for non-ruminants and result in reduced efficiency in fibrous feed utilization compared to ruminants [116].

The fundamental distinction lies in the foregut fermentation strategy employed by ruminants versus the hindgut fermentation approach of non-ruminants, which profoundly influences microbial diversity patterns, energy extraction mechanisms, and host–microbe interactions [122,123]. Ruminant microbiota demonstrate enrichment with fibrolytic and methanogenic communities essential for efficient fiber degradation, whereas non-ruminant microbiota are characterized by predominant saccharolytic and lactate-utilizing taxa that complement enzymatic digestion processes [68,124,125,126]. While these distinctions are well-documented, significant research gaps persist regarding the comparative functional dynamics of these microbial ecosystems under varying dietary regimens and environmental pressures. Advanced metagenomic and multi-omics approaches could further elucidate the co-evolutionary relationships between microbial communities and host physiology, potentially providing insights for targeted dietary interventions and microbiota-based strategies aimed at improving feed efficiency and reducing methane emissions in both ruminant and non-ruminant livestock systems. Table 2 summarizes the predominant microbial groups found in the gastrointestinal tracts of both ruminant and non-ruminant herbivores.

## 5. Metagenomic Technology in Herbivore Gastrointestinal Microbes

Metagenomic technologies have significantly advanced our understanding of microbial communities in herbivores, particularly within their gastrointestinal (GI) tracts. These technologies allow for comprehensive analyses of microbial diversity, functional capabilities, and host–microbe interactions without the need for culturing individual species [145,146,147,148]. Unlike traditional culture-based techniques, which rely on isolating and growing individual microorganisms, metagenomics enables the direct sequencing of all microbial DNA present in environmental samples, such as feces, rumen contents, or intestinal digesta [149,150,151]. This approach allows researchers to capture the genetic material of both culturable and unculturable microorganisms, offering a more holistic view of microbial diversity [152,153]. High-throughput sequencing technologies such as Illumina and Nanopore sequencing generate large volumes of DNA data rapidly, providing insights into the full microbial community composition, including bacteria, archaea, fungi, and even viruses [154,155]. These sequencing methods, combined with advanced bioinformatics tools, facilitate taxonomic classification and functional annotation of microbial genes, enabling researchers to identify key metabolic pathways, microbial interactions, and their potential roles in herbivore nutrition and health.

Furthermore, metagenomic technology is crucial for studying herbivore microbiota’s ability to break down complex plant polysaccharides like cellulose and lignin, enabling the discovery of microbes, enzymes, and metabolic pathways vital for digestion and energy extraction [156,157,158]. The microbial communities in the gastrointestinal tract of herbivores play a pivotal role in fermenting complex substrates, producing short-chain fatty acids (SCFAs) such as acetate, propionate, and butyrate [159,160]. These SCFAs serve as primary energy sources for the host and are crucial for maintaining gut health and metabolic function.

The emergence of high-throughput omics technologies has revolutionized microbiome studies, enabling extensive and large-scale analysis of microbial communities’ structure and function. These cutting-edge techniques have made it possible to explore microbial ecosystems with unprecedented detail, greatly improving our understanding of microbial interactions and their contributions across different environments [161]. Technologies such as metagenomics, metatranscriptomics, metaproteomics, and metabolomics offer a detailed analysis of the genetic, transcriptional, protein, and metabolic characteristics of microbial communities. Unlike conventional techniques, meta-omics enable researchers to examine microbial communities in their native environments, eliminating the need for cultivation and providing a more precise and comprehensive understanding of microbial ecology and function [162].

Metagenomics has emerged as an essential method for examining the microbial communities in ruminants like cattle and sheep, allowing researchers to analyze microbial DNA directly from these animals and explore how changes in these communities correlate with animal traits [163,164,165]. Studies have revealed the intricate interactions between bacteria and archaea in the rumen, especially under conditions where methane mitigation is effective [163,164,165]. Metagenomics has revolutionized the study of microbial ecosystems, particularly in the rumen, by enabling direct DNA sequencing from environmental samples, thereby enhancing our understanding of microbial functions and providing opportunities for targeted mitigation strategies, such as reducing methane emissions in ruminants [166].

Advanced metagenomic techniques, including shotgun sequencing and metatranscriptomics, have enabled the identification of specific enzymes like cellulases and hemicellulases involved in the breakdown of plant polysaccharides. For example, the gut microbiota of Plateau pika revealed that upregulated expression of these enzymes facilitates energy extraction from grass-based diets, particularly at high altitudes [27]. Similarly, metagenomics and metatranscriptomics have revealed the role of Gangba sheep’s gut microbiota in plant biomass degradation, identifying key enzymes involved in the breakdown of plant polysaccharides. These studies highlight how diet and environment influence microbial functions and energy extraction [167]. Using metagenomic and metatranscriptomic sequencing, the rumen microbiota of Gir cattle under different dietary regimes was examined. This approach allowed for the identification of differential microbial populations and their functional dynamics, revealing key transcriptionally active genera like *Caldicellulosiruptor* and *Paludibacter* involved in fiber degradation [168]. Metatranscriptomics is a powerful meta-omics technique that provides insights into the functional dynamics of the rumen microbiome by analyzing RNA transcripts expressed by the microbial community at a given time [169]. Unlike metagenomics, which reveals the genetic potential of the microbiome, metatranscriptomics focuses on identifying the genes that are actively transcribed, offering a real-time view of microbial activity and their functional roles within the rumen ecosystem [170].

Metatranscriptomics serves as a robust tool for analyzing microbial composition and functions related to methane production within individual ruminant species, as well as comparing methane generation differences across various breeds. For example, metatranscriptomic analysis of Holstein cows (low-CH4 emitters) showed increased expression of genes associated with alternative hydrogen disposal pathways, particularly those linked to amino acid synthesis and propionate production [171]. Microbial research faces significant challenges due to the complexity of microbiota and the inherent difficulties in isolating and culturing microorganisms. The advent of metagenomic technology has provided a powerful analytical tool for investigating these complex ecosystems by enabling the sequencing and characterization of all microbial DNA within a sample without requiring individual cultivation [172]. Metagenomics allows researchers to determine microbial composition, identify functional genes, and elucidate metabolic pathways and their products through sophisticated bioinformatics approaches. Herbivore intestinal microbiota not only facilitate feed digestion but also enhance nutrient absorption, synthesize essential vitamins and amino acids, and contribute to immune regulation. These microorganisms degrade complex plant polysaccharides such as cellulose and lignin into SCFAs, which serve as primary energy sources for the host. The gastrointestinal microbiota encompasses bacteria, archaea, fungi, and protozoa, with dominant bacterial phyla including *Bacteroidetes*, *Firmicutes*, *Actinobacteria*, *Proteobacteria*, *Clostridia*, and *Verrucomicrobia* [173,174,175,176,177]. The composition and diversity of these microbial communities are influenced by multiple factors including host age, genetics, diet, environment, and physiological state.

## 6. Microbial Diversity in Herbivore Gastrointestinal Tract

Microbial composition exhibits distinct variation along different sections of the gastrointestinal tract. In ruminant digestive systems, the forestomach harbors the greatest microbial density, followed by the large intestine, with significantly reduced microbial populations in the small intestine [178,179]. Metagenomic studies have revealed distinct microbial patterns along the herbivore gastrointestinal tract. In ruminants, the rumen and stomach are dominated by *Prevotella*, *Fibrobacter*, and unclassified *Bacteroidales* and *Clostridiales* [180], while the small intestine exhibits lower microbial diversity but higher functional activity, with enrichment of *Actinobacteria* and *Patescibacteria* [181]. The large intestine is primarily colonized by *Ruminococcaceae*, *Rikenellaceae*, and *Bacteroidaceae*, facilitating fermentation and energy extraction [182]. Sequencing studies confirm that *Oscillospiraceae*, *Lachnospiraceae*, and *Bacteroidaceae* are dominant bacterial families in the cow rumen, where they drive the essential processes of cellulose degradation and VFA synthesis [183]

In monogastric herbivores, the hindgut, including the cecum and colon, serves as the primary fermentation site. Horses and donkeys demonstrate high abundances of *Firmicutes* and *Bacteroidetes*, with genera such as *Clostridiales*, *Bacteroidales*, and *Ruminococcus* dominating [7,59,184], with *Bacillota* and *Bacteroidota* representing the most abundant phyla. In the newborn goat rumen, *UBA636*, *Bacteroides*, *Rothia*, and *Porphyromonas* species constitute dominant members, although their abundance declines sharply by the tenth day. Consistently Mi et al. [185] demonstrated that most archaeal genomes belong to *Methanobacteriaceae* and *Methanomethylophilaceae*. Furthermore, a study investigated eight different gastrointestinal segments from Bactrian camels, identifying *Firmicutes*, *Verrucomicrobia*, and *Bacteroidetes* as dominant phyla [186]. The diversity index in the rumen was significantly higher than in other segments, while jejunum samples exhibited the lowest richness and diversity. Rabbits are dominated by *Firmicutes*, *Bacteroidetes*, and *Tenericutes*, with *Ruminococcaceae* and *Lachnospiraceae* prevalent at the family level [69].

Environmental and dietary adaptations significantly shape microbial communities in herbivores. Camels harbor *Bacteroidetes* and *Firmicutes*, with genera *Prevotella*, *RC9_gut_group*, and *Butyrivibrio* dominating [53,54]. Drought- or altitude-adapted goats and sheep exhibit increased rumen fungi and shifts in bacterial composition to optimize nutrient utilization under stress conditions [49,186,187,188,189]. Feed interventions such as solid-state fermentation, probiotics, or prebiotics can modulate microbial communities, enhancing beneficial bacteria while suppressing pathogenic species [186,188,189,190,191,192].

Despite diversity and compositional differences in gastrointestinal microbiota among different animal species, *Firmicutes* and *Bacteroidetes* consistently maintain dominant positions and serve as important participants in feed digestion and fermentation. *Proteobacteria* also occupy prominent positions in host microbiomes. This pattern has been confirmed across various animals species, including sheep [193], yaks [194], deer [188], camels [186,189], horses [190,191,192], and rabbits [56], facilitating host adaptation to complex internal environments. Across species, *Firmicutes* and *Bacteroidetes* consistently dominate and play key roles in fiber fermentation, energy production, and host adaptation. Other phyla, including *Proteobacteria* and *Actinobacteria*, contribute to host metabolism and immune regulation. Archaeal genome construction using metagenomes from 10 different ruminants revealed that most archaeal genomes belong to *Methanobacteriaceae* and *Methanomethylophilaceae*. In digestive tract, *Methanobacteriaceae* and *Methanomethylophilaceae* predominate. *Methanobacteriaceae* demonstrated greater abundance in the small intestine compared to the stomach and large intestine, while *Methanobacteriaceae* and *Methanocorpusculaceae* were more prevalent in the large intestine and feces. Overall relative abundance and composition of archaeal genomes vary with species composition.

He et al. [186] investigated eight different gastrointestinal segments from Bactrian camels, identifying *Firmicutes*, *Verrucomicrobia*, and *Bacteroidetes* as dominant phyla. Rumen diversity indices significantly exceeded those of other segments, while jejunum samples exhibited the lowest richness and diversity. *Firmicutes* dominated the entire intestinal microbial community, with *Bacteroidetes* following in the forestomach. *Prevotella*, *Fibrobacter*, unclassified *Bacteroidales*, unclassified *BS11*, and unclassified *Clostridiales* were significantly enriched in forestomach sites. *Firmicutes* and *Verrucomicrobia* represented the most abundant taxa in the ileum and large intestine, with unclassified *Ruminococcaceae* more enriched in the large intestine and ileum than in other gastrointestinal samples. *Proteobacteria* constituted the second most abundant microorganisms in the duodenum and jejunum.

These compositional differences may result from varying feeding conditions. Amplicon sequencing performed on rabbit cecum and fecal samples revealed *Firmicutes*, *Tenericutes*, and *Bacteroidetes* as the primary contributors to microbial diversity. At the family level, *Ruminococcaceae* and *Lachnospiraceae* were predominant [69]. Metabolic processes related to amino acid biosynthesis, energy production, enzyme families, vitamins, and other amino acids demonstrate greater activity in the hindgut compared to the foregut, while carbohydrate metabolism and other amino acid processes show higher activity in the foregut than hindgut, consistent with hindgut fermentation characteristics [184].

Batinah goats coping with drought and water scarcity exhibit increased rumen fungal concentrations [46]. Hu sheep introduced to arid, high-altitude regions demonstrated significant microbiota changes to facilitate environmental adaptation [195]. Solid-state fermented feeds can induce gastrointestinal tract acidification, providing appropriate conditions for beneficial bacterial establishment while reducing *Enterobacteriaceae* and *Salmonella* levels [196,197,198]. *Lactobacillus* supplementation increases relative abundance of the *Firmicutes* phylum while simultaneously decreasing *Bacteroidetes* phylum abundance [197]. Xylitol can promote phosphate acetyl transferase transcription and increase propionate production, thereby reducing pH values to inhibit *Escherichia coli* and *Staphylococcus* growth [198].

### Uncultivated Microbial Lineages in Herbivore Guts

Recent metagenomic studies have significantly advanced our understanding of microbial diversity in herbivore gastrointestinal tracts, revealing novel, uncultivated microbial lineages [199]. Among these are UBA1242 (*Firmicutes*), Rs-D84 (*AlphaProteobacteria*), and UBA9783 (*Verrucomicrobiota*), identified in fecal samples from various farm animals, including cows, yaks, and sheep [200].These lineages are characterized by reduced genomes (<1 Mbp) and the absence of essential biosynthetic pathways, suggesting they rely on metabolites from their hosts, adapting to either a symbiotic or parasitic lifestyle [200]. Notably, UBA9783 possesses a nearly complete glycolytic pathway, indicating its ability to process carbohydrates, while UBA1242 and Rs-D84 exhibit more limited metabolic capabilities, emphasizing the metabolic specialization of these uncultivated taxa [200]. These microbes, particularly in regions such as the four-chambered stomach, contribute to specialized functions like polysaccharide degradation and hydrogen production [199]. The identification of these uncultivated microbial taxa and their associated metabolic pathways highlights the critical role of metagenomics in uncovering previously uncharacterized microbes within the herbivore gut microbiome [199]. These discoveries suggest potential pathways for improving feed efficiency and animal production [200].

## 7. Functional Roles of Gastrointestinal Microbiota in Herbivores and Antimicrobial Resistance (AMR)

The gastrointestinal microbiota of herbivores plays a critical role in nutrient digestion, energy metabolism, and overall host physiology. In ruminants, foregut microbial communities are enriched with genes responsible for degrading complex plant carbohydrates, proteins, and lipids. The small intestinal microbiota primarily focuses on nucleic acid and xenobiotic metabolism, while the large intestine hosts microbial communities dedicated to fermentation and protein synthesis pathways (Table 3) [48,180,181,201,202]. In monogastric herbivores, such as horses and donkeys, hindgut fermentation predominates, with the cecum and colon serving as primary sites of microbial carbohydrate metabolism and SCFA production (Table 4) [203,204,205].

Metagenomic studies have consistently revealed higher abundances of carbohydrate-active enzymes (CAZymes) in the large intestine compared to the small intestine, with GH13 family enzymes playing a significant role in starch degradation [48]. These microbial functions are crucial for breaking down ingested plant material into simple sugars and SCFAs, which serve as major energy sources for growth, development, and maintenance of physiological homeostasis [48,201,203]. These comprehensive metagenomic studies not only enhance our understanding of donkey biology but also establish foundational knowledge applicable to other non-ruminant equines, including horses, while contributing to improved animal health management and disease surveillance strategies [206].

Carbohydrate degradation and SCFA production represent fundamental microbial functions that sustain host energy balance. *Bacteroidetes* species contribute by encoding extensive CAZyme repertoires that degrade complex polysaccharides and host-unutilized glycans, producing acetate and propionate, whereas *Firmicutes*, particularly Ruminococcaceae and Lachnospiraceae, specialize in cellulose and hemicellulose depolymerization and butyrate synthesis [48,201,203,204,205,207,208]. These SCFAs not only supply energy but also support gluconeogenesis, regulate lipid metabolism, and modulate intestinal epithelial function. Comparative metagenomic analyses across sheep, goats, and donkeys demonstrate that carbohydrate, amino acid, lipid, and energy metabolism pathways are broadly represented in both ruminants and monogastric herbivores [48,201,203]. Despite these advances, quantitative mapping of enzyme activity to the actual, final yield of SCFA under varying dietary conditions and host genotypes remains limited, highlighting the need for integrated fluxomics and longitudinal studies [180,181]. There is a notable lack of standardized in vivo assays that link microbial enzyme families to actual SCFA production under different dietary and host genetic conditions.

Beyond nutrient metabolism, gastrointestinal microbiota exert critical immunomodulatory and barrier-protective functions. Microbial metabolites, especially butyrate, regulate tight junction protein expression, mucin secretion, and antimicrobial peptide production, enhancing mucosal integrity and resistance to pathogen colonization [209,210]. Studies in ruminants, such as sheep and goats, have shown positive correlations between the abundance of Bifidobacterium, Ruminococcus, and Enterococcus with mucin gene expression, with metagenomic analyses revealing mucin-degrading enzyme potential in host-adapted taxa [211,212]. Furthermore, probiotic interventions using Bifidobacterium, *Lactobacillus*, and Bacillus have been shown to stabilize microbial communities and reduce enteric disease risk, although strain-specific efficacy and dose–response relationships still require further controlled trials [213,214].

Microbial community composition also significantly influences disease susceptibility and metabolic resilience. Expansions of *Proteobacteria*, which include many pathobionts, are associated with dysbiosis and inflammatory disorders such as colitis, while symbiotic Enterobacteriaceae can occupy inflammatory niches without pathogenic consequences [215,216]. Conversely, *Actinobacteria*, including Bifidobacterium and other taxa (Eggerthellaceae, Nocardiaceae), contribute essential vitamins, amino acids, antioxidants, and bioactive metabolites with antimicrobial and immunomodulatory effects [217]. In both ruminants and non-ruminants, balanced microbial communities dominated by *Firmicutes* and *Bacteroidetes* optimize digestion, SCFA production, and nutrient synthesis, whereas microbial imbalances can disrupt metabolic homeostasis and impair host health [48,201,203,204,205,207,208]. These findings underscore the multifaceted roles of gut microbiota in supporting herbivore nutrition, immunity, and resilience across different digestive system architectures.

The occurrence of AMR genes in herbivore microbiota, particularly in yak, beef, and dairy cattle, significantly affects animal health and production. Studies show that yaks, raised in low-density, antibiotic-free environments, exhibit fewer AMR genes compared to beef and dairy cattle, which are raised in high-density conditions with frequent antibiotic use. This indicates that antibiotics in intensive farming systems contribute to the rise in AMR [218]. Furthermore, mobile genetic elements (MGEs), like integrons, play a vital role in the horizontal transfer of AMR genes. Interestingly, integron abundance was higher in yaks than in beef and dairy cattle, highlighting the role of MGEs even in low-antibiotic environments [218].

In swine, a study by Rahman et al. found that 85.3% of bacterial isolates from the gut microbiota harbored AMR genes, including those for tetracycline, macrolides, and aminoglycosides. The use of whole-genome sequencing (WGS) to analyze 129 isolates helped establish a biobank, aiding in the understanding of MGE involvement in AMR gene transmission identified 246 AMR genes across 38 families, with key resistance genes linked to tetracycline and lincosamide resistance, emphasizing the crucial role of metagenomic tools in AMR monitoring and surveillance [219]. Additionally, research revealed that animals raised with feed additives in barns had a significantly higher AMR profile compared to pasture-raised animals. The resistome in barn-raised cattle was dominated by β-lactamases and tetracycline resistance genes, underscoring the impact of antibiotic use in livestock production. This highlights the importance of integrating metagenomic techniques in AMR surveillance to protect both animal health and public safety [220].

**Table 3:** Major microbial groups, metabolites, and host functions in the gut of ruminant herbivores.

Microbial Group/ Exemplar Taxa	Principal Digestive/ Immune Functions	Predominant GIT Region(s)	Host Effects/ Phenotypes	Representative Hosts	References
*Bacteroidetes* (e.g., *Bacteroides*, *Prevotella*)	Degrade complex plant polysaccharides; utilize host-unabsorbed glycans; contribute to protein/lipid breakdown	Rumen/forestomach; large intestine	Energy harvest; suppression of pathogens; support barrier and immune tone	Cattle, sheep, goats, camels,	[180,181,182,201,203,204,205]
*Firmicutes* (*Ruminococcaceae*)	Degrade resistant polysaccharides, cellulose, and starch; produce degradative enzyme systems	Butyrate	Promote epithelial proliferation, energy harvest, regulate mucosal immunity	Cattle, goats, sheep	[48,201,221]
*Firmicutes* (*Lachnospiraceae*)	Fiber decomposition, protein hydrolysis, butyrate production	Butyrate, secondary metabolites	Support intestinal barrier, promote fat accumulation, gluconeogenesis	Sheep, goats	[207,208]
Phyla: *Bacteroidetes*, *Firmicutes* (>80%); *Proteobacteria*, *Verrucomicrobia*, *Fibrobacteres*, *Spirochaetes*, *Tenericutes*	Polysaccharide breakdown, fiber fermentation, SCFA production, immune modulation	Rumen, caecum, colon	Provide energy via VFAs; shifts with age, diet, and environmental factors; dysbiosis linked with inflammation	Goats	[222,223,224,225]
Families: *Prevotellaceae*, *Veillonellaceae*, *Lachnospiraceae*, *Rikenellaceae*, *Ruminococcaceae*	Fiber degradation, starch fermentation, butyrate production	Rumen, hindgut	Support efficient digestion, gut homeostasis, metabolic flexibility	Goats	[225,226]
*Bacteroidetes* (*Prevotella*, *Bacteroides*)	Fiber degradation, VFA production, carbohydrate	Rumen, hindgut	Improved feed efficiency, energy harvest	Sheep, Tibetan sheep, Mongolian	[227,228,229]
*Firmicutes* (*Ruminococcus*, *Lachnospiraceae*, *Oscillospira*, *Clostridia*, *Lactobacillales*)	Cellulose degradation, butyrate production, gut health maintenance	Rumen, intestine	Correlated with feed efficiency; role in gut homeostasis	Sheep, Qinghai	[230,231,232]
*Bacteroidetes* (*Prevotella*, *Bacteroides*)	Fiber and carbohydrate breakdown, VFA production, carbohydrate metabolism	Rumen, hindgut	Enhanced feed efficiency, energy harvest, gut homeostasis	Cattle (dairy, beef)	[233,234,235]
*Proteobacteria* (*Succinivibrio*, *Acinetobacter*)	Carbohydrate fermentation, starch metabolism	Rumen	Influenced by high-grain diets; amylolytic activity	Cattle	[178,236,237]

**Table 4:** Major microbial groups, metabolites, and host functions in the gut of non-ruminant herbivores.

Microbial Group/Exemplar Taxa	Main Functions	Main Metabolites	Metabolite/Host Functions	Representative Animals	References
*Bacteroidetes* (*Bacteroides* spp.)	Carbohydrate and protein breakdown; enriched in arachidonic acid metabolism, pentose/glucuronate pathways	Acetate, propionate	Provide host energy, enhance barrier, reduce pro-inflammatory cytokines	horse,	[238]
*Firmicutes* (*Ruminococcaceae*)	Cellulose and hemicellulose degradation; resistant polysaccharide breakdown	Butyrate	Promote fat accumulation, energy harvest, barrier support	Donkey	[203,239]
*Firmicutes* (*Lachnospiraceae*)	Fiber decomposition, protein hydrolysis	Butyrate, secondary metabolites	Energy metabolism, gut homeostasis	Donkey, rabbit	[239,240]
Phyla: *Firmicutes*, *Verrucomicrobiota*	Fiber degradation, SCFA production, immune modulatio	Caecum (main fermentation site)	Seasonal abundance variations linked to productivity, physiology, and immune responses	Rabbits	[70]
Genera: *Akkermansia*, *Blautia*, *Oscillospira*	Mucus degradation (*Akkermansia*), fermentation, SCFA production	Soft feces (caecotropes)	Enhanced nutrient recycling via caecotrophy; improved metabolic health	Rabbits	[68]
*Bacteroidetes + Firmicutes interplay*	Co-metabolism of polysaccharides; *Firmicutes* specialize in cellulose fermentation, *Bacteroidetes* in glycan breakdown	Mixed SCFAs (acetate, propionate, butyrate)	Ensure efficient fiber digestion, provide major VFAs for host energy	Donkey, horse	[172,179,241]

## 8. Role of Gastrointestinal Microbiota in Production Performance of Herbivorous Animals

**Figure 4:**
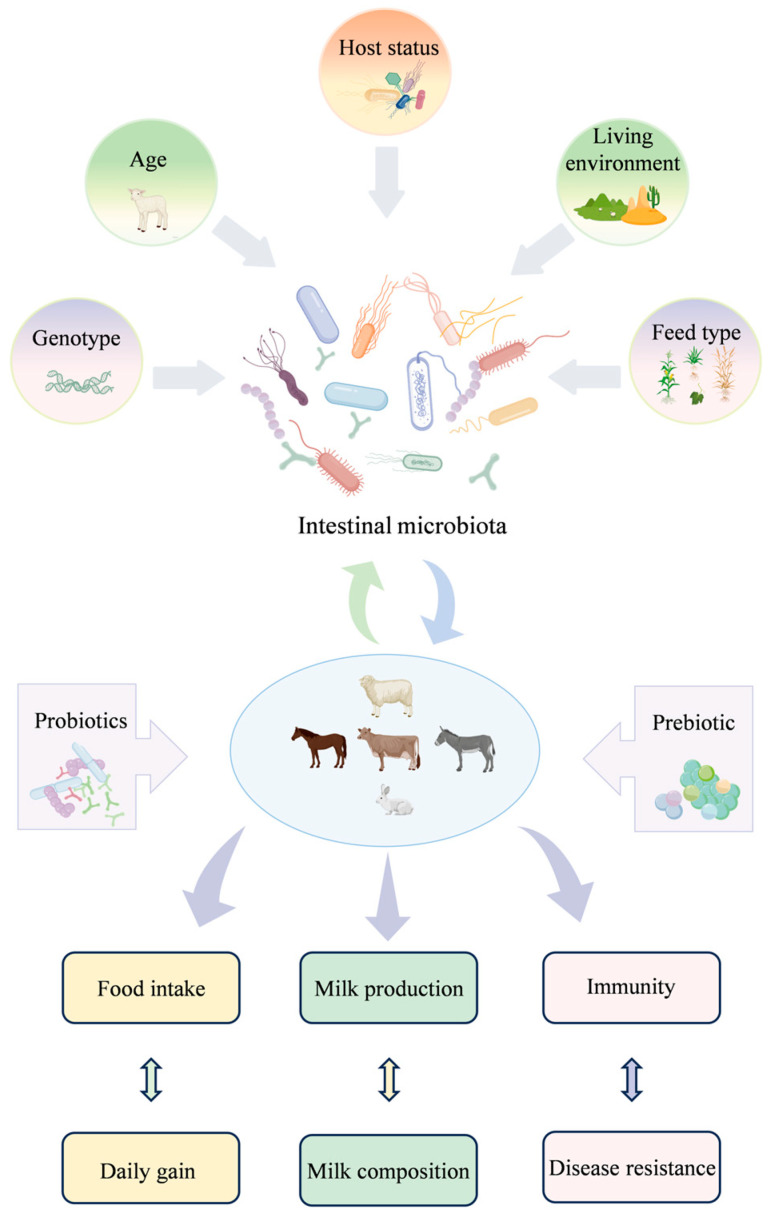
Integrated model of the gastrointestinal microbiota as the metabolic engine of production performance in herbivorous animals.

Herbivorous animals depend on complex gastrointestinal systems and symbiotic microbial communities to efficiently convert fibrous feedstuffs into high-quality animal protein, thereby supporting optimal growth and production performance. The intestinal microbiota plays a central role in nutrient metabolism, immune modulation, and maintenance of intestinal barrier integrity, collectively enhancing feed utilization, daily weight gain, and disease resistance [124,242,243]. Factors such as age, diet, host genetics, and living environment significantly influence the composition and functional diversity of gut microorganisms, as illustrated in Figure 4. In ruminants, the balance between *Firmicutes* and *Bacteroidetes* in the rumen is closely associated with milk fat yield, energy storage, and average daily gain (ADG), while the presence of fibrolytic bacteria such as *Fibrobacter* and *Eubacterium ruminantium* positively correlates with milk and protein production [244,245,246,247]. Conversely, decreased populations of beneficial microbes, including *Bifidobacterium* and *Lactobacillus*, have been linked to reduced production efficiency and compromised animal health [248,249].

Feed efficiency (FE) and residual feed intake (RFI) represent critical indicators of production performance, reflecting how effectively animals convert feed into body mass or milk production. Metagenomic investigations have also revealed that concentrate feeding sequences influence volatile fatty acid production and microbial community composition in both weaned and adult donkeys [250]. The application of metagenomic technology has been particularly valuable in understanding how dietary interventions and physiological conditions modulate the donkey microbiome and subsequent health outcomes. Research utilizing metagenomic analyses has demonstrated that dietary energy levels significantly impact cecal microbial diversity and metabolome profiles, with low-energy diets causing oxidative stress and growth reduction through alterations in microbial metabolite production [251].Recent studies have demonstrated that specific microbial species or strains, rather than entire microbial communities, exert stronger influences on feed efficiency in both ruminants and monogastric animals [252,253,254,255]. For example, *Ruminococcus gauvreauii* abundance is positively associated with dry matter intake (DMI) in dairy cows, whereas *Howardella* correlates with reduced DMI [244,256]. Similarly, microbial populations in the jejunum, cecum, and colon—including *Prevotella*, *Clostridium*, *Oscillospira*, and *Faecalibacterium prausnitzii*—have been shown to influence feed utilization and growth performance in beef cattle [257]. In monogastric herbivores such as donkeys, dietary interventions including corn silage supplementation can modulate the abundance of carbohydrate-metabolizing microbes such as *Prevotella-1* and *Alloprevotella*, thereby improving ADG and overall growth performance [105,258,259,260]. Despite these significant findings, the complex interactions between feed characteristics, energy requirements, and microbial activity remain incompletely understood, highlighting the critical need for additional mechanistic studies.

The strategic application of probiotics, prebiotics, and synbiotics represents a promising approach to enhance gastrointestinal microbial balance and promote animal health and productivity. Prebiotics, including fructooligosaccharides (FOS) and galactooligosaccharides (GOS), selectively stimulate beneficial microbial populations, preventing pathogen colonization and improving nutrient absorption [257,258,259,260,261]. Probiotic supplementation, including *Bacillus subtilis* natto and *Faecalibacterium prausnitzii*, has been demonstrated to enhance ADG, feed efficiency, and immune function while reducing the incidence of gastrointestinal diseases [261,262]. Synbiotic supplementation further supports growth performance by combining the synergistic effects of probiotics and prebiotics on gut microbial composition, immune function, and nutrient metabolism [263,264]. Additionally, metagenomic technology has facilitated the assessment of dietary supplements, such as yeast polysaccharides, multienzymes, and methionine, on gut microbial composition and host immune function [265,266,267].

Collectively, these findings underscore the inseparable relationship between gastrointestinal microbiota and production performance, suggesting that strategic microbial modulation can optimize growth, lactation, and overall health in herbivorous animals. This approach ultimately promotes sustainable animal husbandry practices by maximizing production efficiency while maintaining animal welfare and reducing environmental impacts through improved feed conversion ratios.

## 9. Challenges and Advances in Sampling Gut Microbiota

Studying gut microbiota in large herbivores and monogastric livestock presents significant challenges due to anatomical constraints and limited accessibility. While fecal sampling remains the most commonly employed non-invasive method, it primarily reflects luminal microbial communities and may not accurately represent mucosa-associated bacteria, which are critical for host–microbe interactions [268]. Fecal microbial composition can also be influenced by factors such as transit time, dietary composition, and environmental exposure, potentially introducing biases in microbiota analysis [233,269]. In ruminants, rumen cannulation (fistulation) allows repeated, direct sampling of ruminal contents and is considered the gold standard for rumen microbiota studies [270]. However, this invasive procedure requires surgical intervention and is largely restricted to controlled research settings. Less invasive alternatives, such as oral stomach tubing, often underrepresent particle-associated microbes essential for fiber digestion [271,272]. Post-mortem sampling provides comprehensive access to gut contents and mucosal scrapings but is constrained by rapid microbial shifts following tissue death and the inability to conduct longitudinal studies [273,274]. Endoscopic and biopsy-based approaches enable investigation of mucosa-associated microbiota, yet these techniques are technically challenging in large animals and frequently necessitate anesthesia or surgical procedures, limiting their application in large-scale studies [268,275].

Recent advances in non-invasive sampling techniques have highlighted buccal swabs as a promising alternative for rumen microbiota assessment. Kittelmann et al. [276] demonstrated that buccal swabs can effectively capture bacterial, archaeal, and eukaryotic community structures, while Tapio et al. [277] reported that regurgitated bolus samples exhibit higher similarity to rumen contents compared to buccal swabs, likely due to distinct gingival microbiota composition. Time-course sampling combined with machine learning approaches further indicated that buccal swabs can detect key microbial taxa, although their accuracy depends on collection timing and environmental conditions [278]. More recently, MinION amplicon sequencing has enhanced the resolution and throughput of buccal swab microbiome profiling, enabling improved characterization of rumen microbiomes while maintaining a non-invasive approach [279]. Despite these promising developments, further research is needed to optimize sampling protocols, address site-specific microbial variability, and validate buccal swabs as reliable proxies for direct rumen sampling. This represents a critical research gap in large-animal gut microbiota studies, emphasizing the urgent need for standardized, non-invasive methodologies that balance animal welfare considerations, sampling accuracy, and practical feasibility.

## 10. Conclusions

This review highlights the pivotal role of metagenomics in advancing our understanding of herbivore gastrointestinal microbiota and its profound impact on animal health, feed efficiency, and production performance. By comparing the microbial ecosystems of ruminants and non-ruminants, it is evident that the specialized fermentation processes in the foregut and hindgut result in distinct microbial compositions that directly influence nutrient metabolism and energy extraction. Key microbial taxa, such as *Prevotella*, *Fibrobacter*, and *Ruminococcus*, play critical roles in fiber degradation, while targeted interventions like probiotics and prebiotics offer promising strategies for optimizing microbial balance and improving productivity.

Despite these advances, challenges such as microbial sampling accuracy, functional gene annotation, and understanding the complex interactions between microbiota and host physiology remain. Moving forward, integrating multi-omics approaches and improving sampling methodologies will be essential for a more comprehensive understanding of microbial dynamics. The continued application of metagenomics in herbivore microbiota research holds great potential for enhancing livestock management practices, improving feed efficiency, reducing environmental impacts, and supporting sustainable agricultural systems.

## Figures and Tables

**Table 1:** Gastrointestinal characteristics of ruminants and non-ruminants.

Classification of Herbivores	Species	Family	Structural Characteristics	Adaptive Characteristics	References
Ruminant (Rumen fermenter)	Cattle	Bovidae	Four-chamber stomach, rumen developed	Rumination behavior, the mechanism of urea recycling in saliva	[37,44,45]
	Sheep			Rumen wall papillae are dense and adapted to high fiber roughage	[46,47]
	Goat			Efficient fiber breakdown through rumen microbes Ability to detoxify tannins and secondary plant metabolites	[48,49]
	Deer	Cervidae		Seasonally adjust the composition of rumen microorganisms	[50,51,52]
	Camels	Camelidae	Three-chambered stomach, lacking omasum	A high proportion of high salt-tolerant bacteria in the rumen and high-water reabsorption efficiency in the colon	[53,54]
Non-ruminants (Hindgut fermenter)	Horse	Equidae	Large cecum volume, well-developed colon, short small intestine	Dependent on continuous feeding for fermentation	[55,56,57]
	Donkey		Similar to the horse, the cecum is smaller and the ratio of total length of intestine to body weight is higher than in the horse	The proportion of lignin-resistant bacteria in cecum was higher than that in the horse, which made better use of roughage	[58,59,60]
	Hares	Lepus	The cecum is extremely well developed and the colon is differentiated into a sac-like structure	Secondary digestion is carried out through the act of eating feces	[61,62,63,64,65]
	Rabbit (*Oryctolagus cuniculus*)	Leporidae	Hindgut fermenter; large caecum; produce hard and soft feces (caecotropes)	Caecotrophy for nutrient recycling; seasonal microbiota shifts affect health and productivity	[66,67,68,69,70]

**Table 2:** Predominant microbial groups in the gastrointestinal tract of ruminants and non-ruminants.

Microbial Group	Ruminants	Non-Ruminants	References
Archaea	*Methanobrevibacter* (methanogens)	*Methanobacterium* (methanogens)	[127,128]
	*Methanosarcina* (methanogens)	*Methanobrevibacter* (methanogens)	[128,129]
Bacteria	*Prevotella* (cellulose degradation)	*Bacteroides* (protein and carbohydrate breakdown)	[130,131,132]
	*Fibrobacter* (cellulose degradation)	*Lactobacillus* (fermentation)	[133,134]
	*Ruminococcus* (cellulose degradation)	*Bacteroides* (carbohydrate fermentation)	[135,136]
	*Butyrivibrio* (fiber degradation)	*Clostridium* (carbohydrate fermentation)	[137,138]
Protozoa	*Entodinium* (fiber degradation)	*Holotrichs* (fiber degradation)	[139,140]
Fungi	*Neocallimastix* (fiber degradation)	*Piromyces* (fiber degradation)	[141,142]
	*Anaeromyces* (cellulose degradation)	*Orpinomyces* (cellulose degradation)	[143,144]

## Data Availability

No new data were created or analyzed in this study.
